# Supplementary material for: Raloxifene Suppresses Tumor Growth and Metastasis in an Orthotopic Model of Castration-Resistant Prostate Cancer
Source: Biomedicines. 2022 Apr 5;10(4):853. doi: 10.3390/biomedicines10040853 (PMC9033055; doi:10.3390/biomedicines10040853)
Supplement: Supplementary file 1 [file biomedicines-10-00853-s001.zip › biomedicines-1659447-supplementary.pdf]

biomedicines

MDPI

Supplementary file

Raloxifene suppresses tumor growth and metastasis in an orthotopic model of castration-resistant prostate cancer

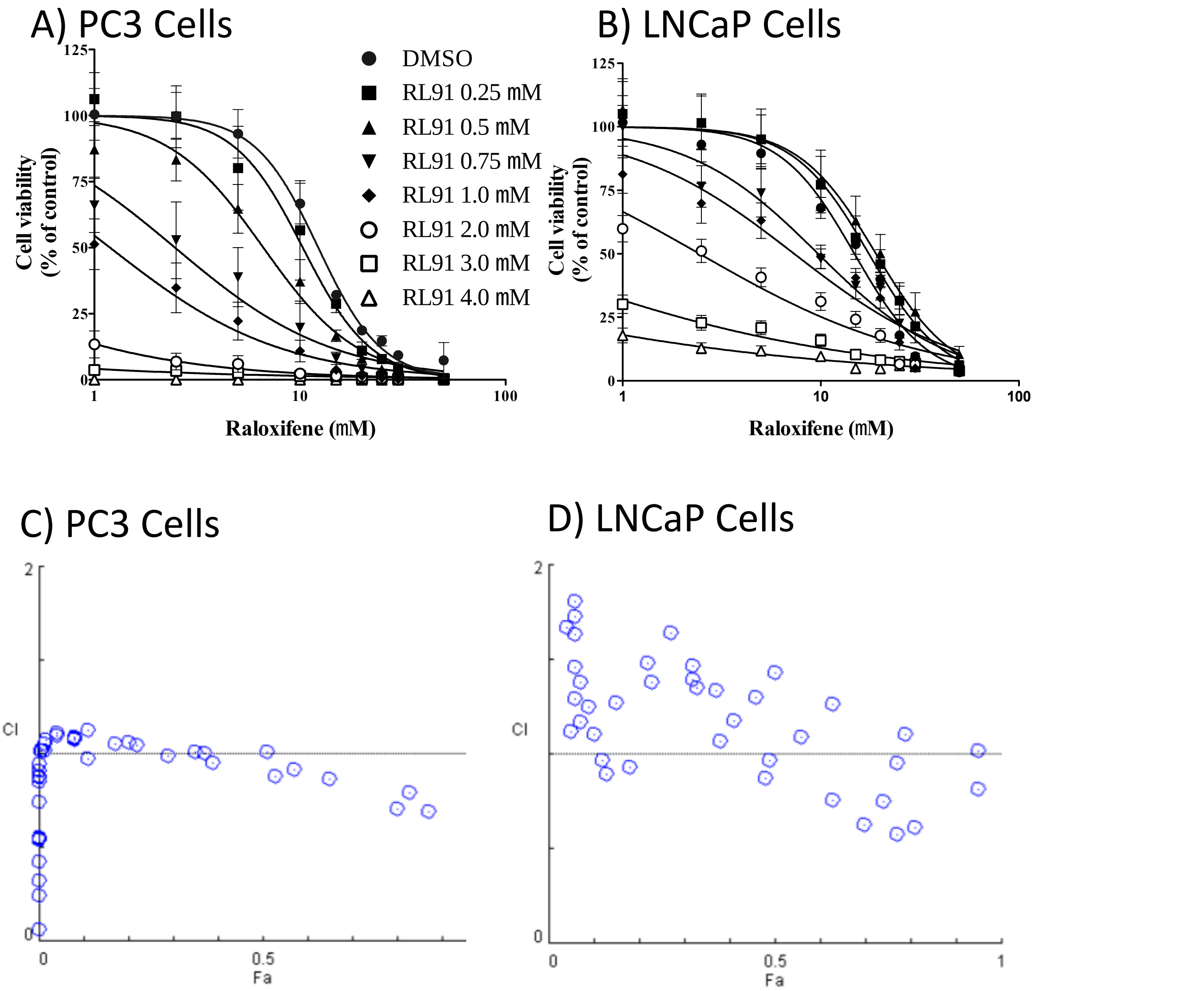

Figure S1. Dose-response of cytotoxicity elicted by raloxifene and RL91 in combination. (A) PC3 cells and (B) LNCaP cells were seeded in 96 well plates at 4x103 and 12x103 cells/well, respectively. Cell number was determined using the SRB assay following 72 h of treatment with raloxifene (1-50 µM) and the addition of RL91 (0.25-4 µM) or a DMSO control. Results are expressed as cell viability as a percentage of control from 3 independent expermints performed in triplicate. Isobologram graphical results showing the combination indexes for each concentraion in combination for (C) PC3 cells and (D) LNCaP cells. Isobolograms were generated using the program CompuSyn.
